# Dibasic Ammonium Phosphate Application Enhances Aromatic Compound Concentration in Bog Bilberry Syrup Wine

**DOI:** 10.3390/molecules22010052

**Published:** 2016-12-29

**Authors:** Shao-Yang Wang, Yi-Qing Li, Teng Li, Hang-Yu Yang, Jie Ren, Bo-Lin Zhang, Bao-Qing Zhu

**Affiliations:** Beijing Key Laboratory of Forestry Food Processing and Safety, Department of Food Science, College of Biological Sciences and Technology, Beijing Forestry University, Beijing 10083, China; wangshaoyang@bjfu.edu.cn (S.-Y.W.); yiqingli@bjfu.edu.cn (Y.-Q.L.); liteng24@126.com (T.L.); yanghangyu2015@163.com (H.-Y.Y.); chxdyd2012@163.com (J.R.); zhangbolin888@163.com (B.-L.Z.)

**Keywords:** bog bilberry syrup wine, dibasic ammonium phosphate, volatile compounds, odor activity value, aroma attributes

## Abstract

A nitrogen deficiency always causes bog bilberry syrup wine to have a poor sensory feature. This study investigated the effect of nitrogen source addition on volatile compounds during bog bilberry syrup wine fermentation. The syrup was supplemented with 60, 90, 120 or 150 mg/L dibasic ammonium phosphate (DAP) before fermentation. Results showed that an increase of DAP amounts accelerated fermentation rate, increased alcohol content, and decreased sugar level. Total phenol and total flavonoid content were also enhanced with the increase of DAP amounts. A total of 91 volatile compounds were detected in the wine and their concentrations were significantly enhanced with the increase of DAP. Ethyl acetate, isoamyl acetate, phenethyl acetate, ethyl butanoate, ethyl hexanoate, ethyl octanoate, ethyl decanoate, isobutanol, isoamyl alcohol, *levo*-2,3-butanediol, 2-phenylethanol, *meso*-2,3-butanediol, isobutyric acid, hexanoic acid, and octanoic acid exhibited a significant increase of their odor activity value (OAV) with the increase of DAP amounts. Bog bilberry syrup wine possessed fruity, fatty, and caramel flavors as its major aroma, whereas a balsamic note was the least present. The increase of DAP amounts significantly improved the global aroma attributes, thereby indicating that DAP supplementation could promote wine fermentation performance and enhance the sensory quality of bog bilberry syrup wine.

## 1. Introduction

Bog bilberry (*Vaccinium uliginosum* L.) has attracted much interest in the field of food and nutritional sciences due to its beneficial health properties [1]. It has been confirmed that these beneficial properties are mainly related to bog bilberry’s high level of phenolic compounds, such as anthocyanins and phenolic acids, since these secondary nutrients possess anti-cancer, anti-cardiovascular, anti-obesity, and anti-oxidative features [2,3]. However, fresh consumption of bog bilberry is not recommended because of its high organic acid-to-sugar ratio compared to other fruits [4]. Sugar fortification has been widely applied to bog bilberry. Its by-product after the process, bog bilberry syrup, has been generally used as the raw material for bog bilberry syrup wine production since it is rich in functional ingredients derived from bog bilberry fruits [5].

Fermentation is a process by which sugar is metabolized by yeasts into alcohol [6]. Therefore, the replication and growth of yeasts during wine fermentation play a significant role in affecting the fermentation performance [7,8]. At the early stage of the fermentation process, nitrogen sources in the fruit matrix play important roles in the growth and replication of yeasts. Sufficient yeast population can provide suitable fermentation activity, which can ensure the sugar-to-ethanol conversion efficiency during the fermentation period [9]. It has been reported that nitrogen supplements, including inorganic nitrogen and organic nitrogen nutrients, have been applied to the fruit wine fermentation process [4,7,8,10,11]. Ammonium salts are the main inorganic nitrogen that is used for wine fermentation, whereas organic nitrogen sources applied to wine fermentation mainly include γ-aminobutryic acid, short peptides, purine, and pyrimidine bases [6]. Compared to organic nitrogen supplements, ammonium salts have been reported to have a more stimulatory effect on the yeast activity [6]. It has been reported that fruit wine fermentation performance could be significantly improved with the supplementation of dibasic ammonium phosphate (DAP) [7,12,13]. For example, the addition of high DAP amounts in grape matrix significantly accelerated the rate of fermentation and enhanced the sugar metabolism, resulting in grape wine with a low residual sugar level [12,14].

Wine sensory quality and nutritional value directly affect its perception and acceptability to consumers [15,16]. Basically, wine sensory quality consists of its appearance, overall aroma, and mouthfeel [17]. Phenolic compounds in wine mainly determine the color attributes and mouthfeel of wine, whereas the overall wine aroma is attributed to the composition and distribution of aromatic compounds in wine [18,19]. It has been accepted that fruit type (raw material), yeast strain, and fermentation conditions play important roles in determination of the overall aroma of final wine products [19]. Moreover, nitrogen supplement has also been reported to impact the aromatic compound composition in wine [10,14,20,21,22,23,24]. For example, it has been reported that nitrogen source type and/or addition amount could regulate the fermentation kinetics, which further altered the composition and profile of aromatic compounds in wines [9,20,21]. An appropriate level of nitrogen source in fruit material can reduce the accumulation of off-flavor volatiles during wine fermentation, improving the sensory attributes of the final wine products after fermentation [10]. To the best of our knowledge, such investigations have only been extensively conducted in grape wine fermentation. Bog bilberry has a totally different aromatic profile compared to grape [25,26]. More importantly, our preliminary study revealed that a stuck fermentation occurred in bog bilberry syrup without the supplementation of DAP, whereas a 30 mg/L DAP addition in bog bilberry syrup resulted in a sluggish fermentation (Supplementary Materials Table S1). These indicated that bog bilberry syrup had a nitrogen source deficiency. Therefore, it is necessary to investigate the effect of nitrogen source supplement (60, 90, 120, and 150 mg/L) on the improvement of sensory attributes in bog bilberry syrup wine after fermentation. The findings from this study could provide practical information on improving the sensory quality of bog bilberry syrup wine.

## 2. Results and Discussion

### 2.1. Physicochemical Indexes

Nitrogen source supplementation is a critical process for fruits with low nitrogen source level or nitrogen deficit before wine fermentation process [9,24]. Our preliminary study showed that a stuck fermentation (more than 30 days) was found in the bog bilberry syrup that was not supplemented with DAP, and its alcohol content was only about 3% (*v*/*v*). Meanwhile, the bog bilberry syrup fermentation under a 30 mg/L DAP supplementation appeared to be sluggish, and the fermented wine only had an about 7% (*v*/*v*) alcohol content (Supplementary Materials Table S1). Such low alcohol contents in these wines reduced their stability and these wines were expected to exhibit a poor sensory feature. A completed fermentation of the bog bilberry syrup with 60 mg/L DAP supplement was achieved in 19 days, whereas 90 mg/L DAP addition caused the bog bilberry syrup wine fermentation to be completed in 17 days. More importantly, higher amounts of the DAP supplement (120 and 150 mg/L) in the bog bilberry syrup significantly shortened the fermentation period to 14 days (Table 1). The density ratio variation of bog bilberry syrup wine fermentation under different DAP amount additions also indicated that higher amounts of DAP (120 and 150 mg/L) resulted in a rapid decrease in the wine density compared with that in the wine treated with 60 mg/L DAP. Meanwhile, the final density of the wine treated by 120 and 150 mg/L DAP were significantly lower than that in the wine with 90 mg/L DAP. These results indicated that DAP exerted a positive effect on the yeast development, which induced the differences of the wine density (Supplementary Materials Figure S1) Our results were similar to a study on grape wine fermentation supplemented with different amounts of DAP [24]. Additionally, a significant increase in the alcohol content was also observed in the bog bilberry syrup wine with the increase in the DAP supplement amount. For example, the alcohol content in the wine with 60 mg/L DAP was 9.1%, whereas the wine with 150 mg/L DAP exhibited the alcohol content at 11.0%. A significant enhancement on the consumption of the total and reducing sugar were also found in the wine with the increase of the DAP amount supplemented in the bog bilberry syrup. Wine pH and acidity affect the wine mouthfeel, and these physicochemical indexes determine the stability of wine [6]. In the present study, the wines supplemented with different amounts of DAP showed similar levels of pH and total acidity level, indicating that the application of the DAP supplement did not significantly impact the mouthfeel caused by wine pH level and acidity.

### 2.2. Total Phenols, Total Flavonoids, Total Anthocyanins, and Color Attributes

Phenolic compounds in wine can be divided into anthocyanins and non-anthocyanin phenolic compounds regarding their contribution to wine sensory quality [27]. Anthocyanins are the most important colorants in wine and play important roles in the appearance of wine, whereas non-anthocyanin phenolic compounds are mainly responsible for the mouthfeel and palatability of wine [18]. Non-anthocyanin phenolic compounds, based on their chemical structures, can be further classified into flavanols, flavonols, and phenolic acids. Table 2 lists the content of the total anthocyanins, total flavonoids, and total phenols of these bog bilberry syrup wines supplemented with different amounts of DAP. No significant effect on the content of the total anthocyanins was found among these different DAP-supplemented bog bilberry syrup wines. However, a significant increase in the level of the total flavonoids and total phenols were observed in these wines with the increase of the DAP supplement amount. For example, the concentration of the total flavonoids in the wine significantly increased from 12.88 mg/L to 14.72 mg/L with the DAP supplement amount from 60 mg/L to 150 mg/L. The wine supplemented with 150 mg/L DAP displayed higher content of the total phenols (892.27 mg/L) compared to the wine with 60 mg/L DAP (749.72 mg/L). It has been shown that oxidation occurs during the wine fermentation process, resulting in the decrease of phenolic compound content [28]. The DAP supplementation in the present study, however, significantly reduced the bog bilberry syrup wine fermentation period, which might prevent phenolic compounds from oxidation since a higher phenolic compound degradation has been reported to be found in a longer fermentation period [29]. Higher content of phenols in wine has been reported to improve the volatility of odorants, which might enhance the release of volatiles from wine and increase the wine sensory perception [30] In addition, non-anthocyanin phenolic compounds have been reported to play important roles in stabilizing anthocyanins during wine fermentation by interacting with anthocyanins via co-pigmentation and polymerization [31]. No significant alteration of the total anthocyanin content was observed in these wines supplemented with different DAP supplement amounts, indicating that the level of phenolic compounds in these wines might be sufficient for the stabilization of anthocyanins.

Regarding the CIELab assay, a* represents the redness/greenness of wine, whereas b* indicates the yellowness and blueness. The lightness of wine is determined by the L* value [32]. It was observed that the wines supplemented with different amounts of DAP exhibited the similar b* value, indicating that the DAP supplementation did not significantly affect the blueness of the wine after the fermentation (Table 2). A fluctuation in the L* value was observed in the wines supplemented with different amounts of DAP. A dramatic impact was observed on the a* value of the wine with different amounts of DAP supplemented in the wine. For example, the a* value significantly increased in the wine supplemented with DAP from 60 mg/L to 120 mg/L. However, the addition of 150 mg/L DAP resulted in a significant decrease in the a* value of the wine. Yeast metabolism has been confirmed to produce acetaldehyde, pyruvic acid, and vinyl phenol. These metabolites have been reported to react with anthocyanins to yield stable pigments in wine, which resulted in an increase in the a* value [21]. The decrease in the a* value in the wine supplemented with 150 mg/L DAP might result from the adsorption of the yielded stable pigments on the yeast cell wall. These results indicate that a relatively higher DAP supplementation might shift the wine color towards red, but a much higher content brought opposite effect, which was not consistent with the pervious study [21].

### 2.3. Volatile Compounds

Volatile compounds in wine include the volatiles released from fruits, the volatiles synthesized during alcoholic fermentation, and the aromatic compounds accumulated at the aging period [6,33]. These volatile compounds determine the overall aroma of wine. Red Fruit^®^ yeast was used in the present study. This commercial yeast strain has been widely used in wine fermentation and it can result in the pleasant blueberry, blackberry, cherry, raspberry and violet aromas with a soft and fresh taste. In the present study, a total of 91 volatile compounds were detected in the bog bilberry syrup wines supplemented with different amounts of DAP. These volatiles included 34 esters, 18 alcohols, five aldehydes, two ketones, ten acids, 16 terpene derivatives, and six other volatile compounds (Table 3).

#### 2.3.1. Esters

Esters are mainly formed during the wine fermentation process under multiple enzymatic reactions [46]. Therefore, the production rate of esters in wine is essentially determined by the population and activity of yeasts [19]. Regarding their molecular and synthetic feature, esters in wine can be further divided into acetate esters, ethyl esters, and other esters [34]. It is known that esters are the major volatile compounds that contribute the fruity and floral notes to the overall aroma of wine [47].

Acetate esters are synthesized from the metabolism of lipids and amino acids linked with acetyl-CoA under a series of enzymatic reactions and different yeast strains and esterase levels significantly affect the distribution of acetate esters in wine [19,48,49]. In this study, acetate esters were the major ester group in the bog bilberry syrup wine (Table 3). The wine supplemented with 150 mg/L DAP showed the highest acetate esters level compared to other wines. Additionally, ethyl acetate, isoamyl acetate, and phenethyl acetate were the predominant individual acetate esters in these wine samples. These acetate esters represented more than 99% of the total acetate ester content (Table 3). Regarding their flavor notes, ethyl acetate provides wine with the pineapple, fruity, solvent, and balsamic aroma, whereas isoamyl acetate exhibits the banana, fruity, and sweet note to wine. Phenethyl acetate has been reported to contribute to wine with the flowery flavor [35]. The concentration of ethyl acetate and isoamyl acetate in the wine samples were higher than their thresholds, indicating that these volatiles could significantly contribute their flavor notes to the overall aroma of the wine. The wine supplemented with 150 mg/L DAP showed much higher ethyl acetate concentration compared to other wine samples. The similar enhancement was also found on the content of isoamyl acetate in the wine with 150 mg/L DAP. Additionally, the increase in the DAP amount resulted in a significant enhancement on the accumulation of phenethyl acetate in the wine after the fermentation. However, its OAV value in these wines was below 1, indicating that the flavor note of phenethyl acetate might not significantly contribute to the overall aroma of the bog bilberry syrup wine. Our results were consistent with the previous studies where acetate esters showed a significant increase in the content in wine supplemented with nitrogen resource [10]. We speculated that the addition of high DAP amount (150 mg/L) in the bog bilberry syrup before the fermentation could stimulate the growth of the yeasts, which could activate the expression of alcohol acetyltransferases in the yeasts. These eventually led to the formation of more acetate esters in the wine [46,50].

The major ethyl esters in the wine samples included ethyl hexanoate, ethyl lactate, ethyl octanoate, ethyl 2-hydroxy-4-methylpentanoate, ethyl decanoate, diethyl succinate, ethyl 9-decenoate, ethyl dodecanoate, and ethyl myristate. Ethyl esters are biosynthesized from ethanol and fatty acids and these esters also contribute unique flavor notes to the overall aroma of wine [34,51]. Although numerous ethyl esters were yielded in these wine samples after the fermentation, only some of them showed their OAV values above 1 (Table 3). The addition of DAP in the bog bilberry syrup wine also showed the effect on enhancing the accumulation of these esters in the wine (Table 3). For example, ethyl butanoate is known to have the strawberry, apple, and banana notes [35]. The ethyl butanoate level in the wine supplemented with 60 mg/L DAP started to be higher than its threshold, and its highest level was observed in the wine with 150 mg/L DAP. Similarly, ethyl hexanoate showed higher OVA value in the wine with 90 mg/L DAP, which could provide the wine with a better aroma with the fruity, green apple, banana, brandy, and wine-like aroma notes [35]. Additionally, the OAV value of ethyl octanoate was higher than 1 in the wine supplemented with 150 mg/L DAP (Table 3). However, its concentration was below its threshold in the other wine samples. This indicated that the flavor notes of ethyl octanoate (sweet, floral, fruity, banana, pear, and brandy) could be incorporated into the overall aroma of the wine supplemented with 150 mg/L DAP. The OAV value of ethyl decanoate also significantly increased with the increase of DAP amount in the wine samples, which was consistent with that in grape wine supplemented with nitrogen sources [51]. This indicated that the brandy, fruity, and grape aroma notes derived from ethyl decanoate could benefit the DAP-supplemented bog bilberry syrup wine aroma [35].

Fatty esters of high alcohols (other esters) also showed a significant concentration increase in the wine with the increase of the DAP amount supplemented in the wine (Table 3). However, these esters did not have their OAV values above 1, indicating that their flavor notes contribution was limited.

#### 2.3.2. Higher Alcohols

Higher alcohols are considered the intermediate compounds during the synthesis and catabolism of amino acids [19]. In wine, they are yielded from the metabolism of aldehydes, reductive denitrification of amino acids, and/or synthesis from sugars [19]. It has been accepted that appropriate levels of higher alcohols in wine could bring to the wine a desirable complexity, whereas excessive amounts of these alcohols (above 400 mg/L) negatively affect the overall aroma of wine [52]. In the present study, these wine samples all exhibited the content of the total higher alcohols above 400 mg/L, indicating that these higher alcohols might weaken the overall aroma of the bog bilberry syrup wine (Table 3). The highest content of higher alcohols was observed in the wine supplemented with 90 mg/L DAP, which was in accordance with a previous study where 90 mg/L yeast-assimilable nitrogen supplement caused a dramatic accumulation of higher alcohols in wine [22].

Regarding individual higher alcohols, *levo*-2,3-butanediol, *meso*-2,3-butanediol, 2-phenylethanol, isoamyl alcohol, and isobutanol were the predominant higher alcohols in these wines (Table 3). These major higher alcohols had their OAV values above 1 (Table 3). Both *levo*-2,3-butanediol and *meso*-2,3-butanediol display the fruity, sweet, and buttery notes to the wine aroma, and these higher alcohols are synthesized from acetoin reduction catalyzed by yeasts [35,53]. Their concentration was found to be the highest in the wine supplemented with 90 mg/L DAP. In addition, 2-Phenylethanol, isoamyl alcohol, and isobutanol are formed under the Ehrlich pathway, and their content in the wines showed an obvious increase with the increase of the DAP amount supplemented in the wine (Table 3). In terms of their flavor notes, 2-phenylethanol exhibits the rose and honey aroma, whereas isoamyl alcohol and isobutanol can provide wine with the solvent, sweet, alcohol, and nail polish notes [35]. Therefore, these flavor notes could be more strengthened in the bog bilberry syrup wine with high DAP amount addition. The other higher alcohols were also more accumulated in the wines with the increase of DAP amount. However, their concentration in the wine was below their thresholds, limiting their flavor contribution to the overall aroma of the bog bilberry syrup wine.

#### 2.3.3. Acids

Increasing the amount of DAP resulted in an increase in the content of the total volatile acids in the wine (Table 3). During wine fermentation, the yeast metabolism can produce acetaldehyde. Meanwhile, the addition of DAP has been reported to stimulate yeasts to produce more NADH, which could further oxidize the produced acetaldehyde into acetic acid [54]. Additionally, saturated fatty acid metabolisms can result in the synthesis of higher volatile aliphatic acids, whereas branched chain fatty acids can be generated from the aldehyde oxidation [19].

The major individual volatile acids in the wine samples included isobutyric acid, 2-methylbutanoic acid, hexanoic acid, and octanoic acid. Isobutyric acid, hexanoic acid, and octanoic acid exhibited their content in the wine higher than their thresholds (Table 3). These volatile acids have been reported to possess the rancid, butter, cheese, and fatty notes [35,42], and their OAV values were enhanced in the wine samples with the increase of the DAP amount. Other previous studies reported that these volatile acids showed a decrease in their content in wine as an increase of nitrogen source amount was applied to the grape wine-making process [21,55]. We speculated that the DAP supplementation in the present study effectively promoted the growth and replication of yeasts at the early stage of the fermentation process, which allowed the yeasts to efficiently activate their metabolisms on amino acids and thus produced more volatile acids in the wine after the fermentation [56].

#### 2.3.4. Aldehydes and Ketones

Aldehyde precursors can be produced from the oxidation of unsaturated fatty acid and lipoxygenase catalysis. These precursors can further interact with alcohol during fermentation to yield aldehydes in wine [57,58]. Aldehydes can bring the green and grass notes to the wine aroma [19]. In the present study, the increase in the DAP amount resulted in the content increase of the aldehydes in these bog bilberry syrup wine samples (Table 3). However, the OAV values of these aldehydes were below 1, indicating that their flavor contribution to the overall aroma of wine was negligible.

During fermentation, activated fatty acids can be further condensed, which can lead to the formation of ketones in wine [33]. The wine sample supplemented with high amount of DAP showed higher concentration of the ketones, such as 2-undercanone (Table 3). However, their low OAV values indicated that these ketones might not significantly contribute to the overall aroma of the bog bilberry syrup wine.

#### 2.3.5. Terpene Derivatives

Isoprenyldiphosphate and dimethylallyldiphosphate are the major precursors to synthesis terpenes and a further structure rearrangement, oxidation, reduction, and/or hydration can produce numerous terpene derivatives in fruits [26]. Afterwards, terpenes and terpene derivatives can further be conjugated with sugar moiety in fruits to yield bound terpenes and terpene derivatives [19]. Bound terpenes are flavorless and cannot contribute to the overall aroma of wine [19,52]. However, hydrolysis can release free terpenes and terpene derivatives from their bound forms, making these volatiles further improve the flavor aroma to wine [52,59]. In the present study, *trans*-nerolidol, *p*-menth-1-en-8-ol, β-*cis*-farnesene, citronellol, 2,3-dihydrofarnesol, and 4-terpineol were the dominant terpene derivatives in the wine samples (Table 3). The increase of the DAP amount significantly enhanced their concentration in the wine. We speculated that the increase of the DAP amount during the wine fermentation elevated the activity of glycosidases in yeasts, which promoted the hydrolysis of bound terpene derivatives to release more free terpene derivatives [33,60]. However, these terpene derivatives had their OAV values below 1, indicating a negligible contribution to the overall aroma of the wine.

#### 2.3.6. Other Volatile Compounds

Other volatile compounds in the wine included volatile lactone, aromatics, furanones, sulfur compounds, and carbonyl compounds (Table 3). The increase of the DAP amount resulted in an increase in the content of these compounds in the wine, which was consistent with the published study [13]. However, the flavor contribution of these volatile compounds was limited due to their low OAV values in the wine.

### 2.4. Global Aroma Attributes

**Figure 1:**
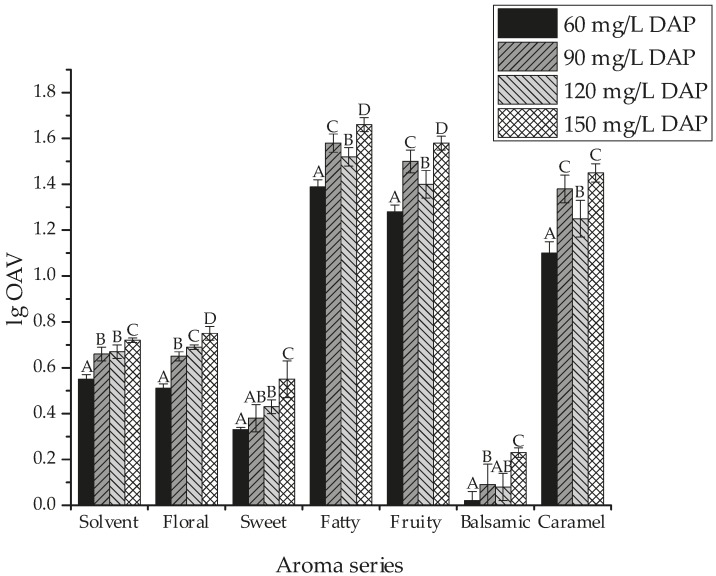
Aroma attributes for the bog bilberry syrup wines supplemented with different amounts of dibasic ammonium phosphate (DAP). Each bar is expressed as the log_10_ transformation of odor activity values (OAV) (sum of corresponding aroma series). Different letters in each series indicate significant differences at *p* ≤ 0.05.

Figure 1 shows the global aroma attributes (log_10_ transformation) of the bog bilberry syrup wines supplemented with different amounts of DAP using the total OAV value summed from each volatile in each given aroma series (Supplementary Materials Table S2). It was observed that fatty, fruity, and caramel aroma were the major aromatic attributes of the bog bilberry syrup wine, whereas the balsamic note was the least present flavor feature. More importantly, the increase in the DAP amount resulted in an obvious enhancement in all the aromatic attributes. For example, the wine with 150 mg/L DAP had the strongest floral flavor, followed by the wine with 120 mg/L DAP, 90 mg/L DAP and then 60 mg/L DAP. The balsamic note was significantly improved in the wine with 150 mg/L DAP compared to the wine with 60 mg/L DAP. These indicated that the sufficient DAP supplementation in bog bilberry syrup before the fermentation process could benefit the overall aroma and complexity of bog bilberry syrup wine.

## 3. Materials and Methods

### 3.1. Chemicals and Reagents

Bog bilberry syrup was received from Xinganlieshen Original Product Ltd., (Hulunbeir City, Inner Mongolia, China). The syrup has a sugar content of 78.1° Brix. Dibasic ammonium phosphate (DAP) was purchased from Fengchuan Chemical Reagent Technologies Co., Ltd., (Tianjin, China). External volatile compound standards included ethyl acetate, isobutyl acetate, phenethyl acetate, ethyl butanoate, ethyl hexanoate, ethyl lactate, ethyl octanoate, ethyl nonanoate, ethyl decanoate, diethyl succinate, ethyl dodecanoate, isoamyl hexanoate, isobutanol, 1-butanol, isoamyl alcohol, 4-methyl-1-pentanol, 1-hexanol, 1-heptanol, 2-nonanol, *levo*-2,3-butanediol, 1-octanol, 1-decanol, benzyl alcohol, 2-phenylethanol, *meso*-2,3-butanediol, 1-dodecanol, nonanal, decanal, acetic acid, isobutyric acid, butyric acid, hexanoic acid, octanoic acid, n-decanoic acid, linalool, *p*-menth-1-en-8-ol, naphthalene, citronellol, geranylacetone, trans-nerolidol, acetoin, *p*-cymene. These standards were purchased from Sigma-Aldrich (St. Louis, MO, USA) with a purity above 95%.

### 3.2. Bog Bilberry Syrup Wine Fermentation

Bog bilberry syrup wine fermentation followed our previously published method [5]. In brief, the bog bilberry syrup was diluted using potable water to a sugar content of 20° brix. Afterwards, the diluted bog bilberry syrup was divided into four groups and supplemented mixed with 60, 90, 120, and 150 mg/L dibasic ammonium phosphate (DAP), respectively, and then transferred to 2.5-L stainless steel fermentation vessels. Subsequently, activated Saccharomyces cerevisiae yeasts (200 mg/L, Red Fruit^®^, Enartis, Italy) were inoculated into the vessel to initiate fermentation. The fermentation was conducted at 20 ± 3 °C and the wine density was monitored using hydrometers. When the relative density of the bog bilberry syrup wine remained consistent for 3 days, K_2_S_2_O_7_ solution (100 mg/L) was added to the vessel to terminate the fermentation. Each treatment was conducted in triplicate.

### 3.3. Physicochemical Indexes

Physicochemical indexes of the bog bilberry syrup wine samples, including alcohol, total sugar, reducing sugar, pH, and total acidity, were measured using the standard chemical analysis methods issued by the National Standard of the People’s Republic of China GB/T 15038-2006 [61].

### 3.4. Total Phenols, Total Flavonoids, Total Anthocyanins, and Color Attributes

The content of total phenols was determined using Folin-Ciocalteu method with minor modifications [62]. Briefly, the wine sample (0.1 mL) was mixed with 0.1 mL of Folin-Ciocalteu reagent and 2.8 mL of distilled water. The resultant mixture was incubated in the darkness for 8 min. Afterwards, the mixture was mixed with 2 mL of 7.5% (*v*/*v*) sodium carbonate solution, and then kept in the darkness for 2 h. The absorbance of the mixture was recorded at 765 nm on a spectrophotometer (Unico Instrument Co. Ltd., Shanghai, China). Gallic acid was used as the external standard, and the content of the total phenols in wine sample was expressed as mg gallic acid equivalents per liter of wine (mg GAE/L). Each measurement was carried out in triplicate.

Total flavonoid content determination followed an aluminum chloride assay with minor modifications [63]. Briefly, 5 mL of 30% (*v*/*v*) wine sample was mixed with 0.3 mL of 5% (*w*/*v*) NaCO_3_ solution. The resulting solution was kept for 6 min. The mixture was mixed with 0.3 mL of 10% (*w*/*v*) Al(NO_3_)_3_ solution and then kept for another 6 min. The resultant mixture was then mixed with 4 mL of 1 mol/L NaOH solution and 0.4 mL of distilled water and incubated at room temperature for 15 min. The absorbance of the mixture was recorded at 510 nm on the same spectrophotometer. Distilled water was used as the blank. Rutin was used as the external standard, and the total flavonoid content of the wine sample was expressed as mg rutin equivalents per liter of wine (mg·rutin/L). Each determination was performed in triplicate.

Total anthocyanin content in bog bilberry syrup wine was assayed using the pH differential method [64]. In brief, the bog bilberry syrup wine (1 mL) adjusted the pH to 1.0 and 4.5 using acetate buffer solution. Then, the absorbance of the resultant sample was calculated using

A (absorbance) *=* (A^pH1.0^_520 nm_ − A^pH1.0^_700 nm_) − (A^pH4.5^_520 nm_ − A^pH4.5^_700 nm_). (1)

The total anthocyanin content was calculated using the equation below and expressed as mg cyanidin-3-*O*-glucoside equivalents per liter of wine (mg Cyanidin-3-*O*-glucoside/L):

Total anthocyanin content (mg/L) = (A × MW × DF × 1000)/(ε × 1)(2)

where MW is the molecular weight of cyanidin-3-*O*-glucoside (449.2 g/mol), DF represents dilution factor, ε is the molar extinction coefficient (26,900 L × mol^−1^ × cm^−1^), and “1” indicates the light length path (cm). Each measurement was conducted in triplicate.

CIELab space method was used to determine the color attributes of the bog bilberry syrup wine samples [4]. The wine sample was filtered through a 0.45 µm filter membrane and the transmittance at 440, 530 and 600 nm were determined using a UV-VIS spectrophotometer (Unico Instrument Co. Ltd., Shanghai, China). Distilled water was used as the blank.

### 3.5. Volatile Compounds

Volatile compounds in the bog bilberry wine were immediately extracted at the end of the fermentation. Solid-phase micro-extraction of volatile compounds from the bog bilberry syrup wine samples followed our published method with minor modifications [65]. In brief, the wine sample (5 mL) was mixed with 1 g NaCl in a 15 mL polytetrafluoroethylene-silicon septum-capped vial containing a magnetic stirrer. The vial was heated at 40 °C for 30 min under agitation. Afterwards, a 50/30 µm DVB/Carboxen/PDMS fiber (Supelco, Bellefonte, PA, USA) was inserted into the headspace of the vial and absorbed volatile compounds in the headspace for 30 min. Finally, the fiber was removed from the headspace of the vial and inserted into the GC injection port at 250 °C for 25 min to desorb the volatiles from the fiber. An HP-INNOWAX column (60 m × 0.25 µm, 0.25 µm sickness, J & W Scientific, Santa Clara, CA, USA) was used for the separation of volatile compounds on Agilent 6890 gas chromatogram (Agilent Technologies, Santa Clara, CA, USA), and analyzed on Agilent 5975 mass spectrometry (Agilent Technologies, Santa Clara, CA, USA). The flow rate of carrier gas (helium) was 1 mL/min, whereas the oven temperature program was as follows: 40 °C for 5 min; rising to 200 °C at a rate of 3 °C/min; and 200 °C for 2 min. GC-MS interface was set at 280 °C and electron impact mode was set at 70 eV. A selective ion mode was used with a mass scan range of *m*/*z* 20 to 450. Identification of volatile compounds was achieved by matching their mass spectrum with NIST08 Mass Spectral Library and further confirmed by their reference standard. Volatile compounds without available reference standard were tentatively identified by comparing their mass spectrum with the Standard NIST 08 library and retention indices with the NIST Standard Reference Database [66]. Volatile compounds were quantified using their external standards calibrated by the internal standard (4-methyl-2-pentanol). Regarding the quantitation of the volatiles without the reference standard, it was quantified by estimating the calibration curve of the available standard with the most similar chemical structure and/or similar carbon atom numbers.

### 3.6. Sensory Attributes and Aromatic Series

Odor activity value (OAV) refers to the sensory contribution of individual volatile compound to the overall aroma of wine [65]. OAV was calculated using the equation below,

OAV = concentration of volatile compound/odor threshold of volatile compound(3)

### 3.7. Statistical Analysis

Data were expressed as the mean ± standard deviation of triplicate tests. One-way analysis of variance (ANOVA) was used to gauge the significant differences among the means under Duncan’s multiple range test at a significant level of 0.05 (SPSS Statistics 23, SPSS Inc., Chicago, IL, USA).

## 4. Conclusions

In conclusion, the increase of the dibasic ammonium phosphate (DAP) amount in bog bilberry syrup accelerated wine fermentation with a high alcohol and phenolic content and a low residual sugar level in wine. Meanwhile, the pH and total acidity of wine were not affected by the amounts of DAP supplement. Almost all the volatile compounds increased their concentration in wine with the increase of the DAP amount during fermentation. Additionally, seven volatile esters, five volatile alcohols, and three volatile acids exhibited high odor activity value in wine, and their odor activity value also significantly increased with the DAP supplementation. Bog bilberry syrup wine fermented under DAP supplementation exhibited fatty, fruity, and caramel flavors as its major aroma, and the increase of DAP amount potentially improved the global aroma attributes of bog bilberry syrup wine.

## Figures and Tables

**Table 1:** Physicochemical indexes of bog bilberry syrup wine supplemented with different amounts of dibasic ammonium phosphate (DAP).

Physicochemical Index	Wine with Dibasic Ammonium Phosphate (mg/L) ^a^			
	60	90	120	150
Fermentation Period (days)	19	17	14	14
Alcohol (%, vol)	9.1 ± 0.2 a	9.8 ± 0.1 b	10.6 ± 0.2 c	11.0 ± 0.1 d
Total Sugar (g/L)	18.84 ± 0.11 c	10.11 ± 0.43 b	10.10 ± 0.17 b	8.32 ± 0.31 a
Reducing Sugar (g/L)	16.07 ± 0.39 c	7.25 ± 0.54 b	7.22 ± 0.03 b	5.84 ± 0.32 a
pH	3.14 ± 0.01 a	3.14 ± 0.01 a	3.14 ± 0.01 a	3.14 ± 0.01 a
Total Acidity (g/L)	8.47 ± 0.17 a	8.66 ± 0.21 a	8.56 ± 0.02 a	8.71 ± 0.07 a

^a^ Data are the mean ± standard deviation of triplicate tests. Different letters in each row indicate significant differences at *p* ≤ 0.05.

**Table 2:** Total anthocyanins, total flavonoids, total phenols, and color attributes of bog bilberry syrup wine supplemented with different amounts of dibasic ammonium phosphate (DAP).

Content	Wine with Dibasic Ammonium Phosphate (mg/L) ^d^			
	60	90	120	150
**Phenolic compounds**				
Total anthocyanins (mg/L) ^a^	36.86 ± 0.30 a	35.36 ± 0.09 a	36.24 ± 1.17 a	38.22 ± 3.65 a
Total flavonoids (mg/L) ^b^	12.88 ± 0.15 a	14.26 ± 0.01 b	14.51 ± 0.13 bc	14.72 ± 0.16 c
Total phenols (mg/L) ^c^	749.72 ± 24.67 a	757.42 ± 33.51 a	836.41 ± 22.24 b	892.27 ± 33.51 b
**Color attributes**				
L* value	47.93 ± 1.15 ab	46.11 ± 0.96 a	46.39 ± 1.05 ab	48.68 ± 0.50 b
a* value	66.74 ± 1.54 b	82.94 ± 1.00 c	81.00 ± 1.04 c	61.60 ± 0.73 a
b* value	−5.40 ± 1.13 a	−6.72 ± 0.71 a	−6.89 ± 1.07 a	−4.75 ± 0.56 a

^a^ Total anthocyanins content is expressed as mg cyanidin-3-*O*-glucoside equivalents/L of wine (mg cyanidin-3-*O*-glucoside/L); ^b^ Total flavonoids content is expressed as mg rutin equivalents/L of wine (mg·rutin/L); ^c^ Total phenol content is expressed as mg gallic acid equivalents/L of wine (mg·GAE/L); ^d^ Data are the mean ± standard deviation of triplicate tests. Different letters in each row indicate significant differences at *p* ≤ 0.05.

**Table 3:** Concentration, odor threshold, and aroma descriptor of individual volatile compounds in bog bilberry syrup wine supplemented with different amounts of dibasic ammonium phosphate (DAP).

Volatile Compound ^a^	Concentration of Volatile in Wine with DAP (mg/L) ^b^				Odor Threshold (mg/L)	Aroma Descriptor
	60	90	120	150		
**Esters**						
Acetate esters						
Ethyl acetate (mg/L) ^A^	**12.88 ± 1.08 a**	**15.55 ± 3.06 a**	**14.92 ± 2.05 a**	**20.70 ± 1.14 b**	12.3 [34]	Pineapple, fruity, solvent, balsamic [35]
Isobutyl acetate ^A^	16.54 ± 0.71 a	21.94 ± 4.47 b	21.42 ± 3.31 ab	30.94 ± 3.27 c	1.6 [34]	Banana, fruity, sweet [34]
Isoamyl acetate ^A^	**258.32 ± 16.06 a**	**251.02 ± 52.85 a**	**279.42 ± 34.35 ab**	**354.55 ± 73.61 b**	0.16 [36]	Banana, fruity, sweet [35]
Octyl acetate ^B^	0.06 ± 0.01 a	0.08 ± 0.01 ab	0.09 ± 0.01 bc	0.10 ± 0.01 c		
Phenethyl acetate ^A^	93.98 ± 5.23 a	163.75 ± 7.57 b	**229.35 ± 15.36 c**	**293.72 ± 29.85 d**	1.8 [36]	Flowery [35]
*Total acetate esters* (mg/L)	13.25 ± 1.01 a	15.99 ± 3.04 a	15.45 ± 2.05 a	21.38 ± 1.18 b		
Ethyl esters						
Ethyl butanoate ^A^	**39.34 ± 4.02 a**	**49.04 ± 11.56 ab**	**40.9 ± 6.85 ab**	**52.39 ± 5.04 b**	0.02 [34]	Strawberry, apple, banana [35]
Ethyl-2-methylbutyrate ^B^	13.34 ± 2.65 a	12.92 ± 2.61 a	14.11 ± 1.32 a	15.44 ± 2.57 a		
Ethyl hexanoate ^A^	**157.75 ± 6.77 a**	**215.04 ± 31.92 b**	**196.54 ± 30.32 ab**	**205.95 ± 44.44 ab**	0.08 [36]	Fruity, green apple, banana, brandy, wine-like [35]
Ethyl heptanoate ^B^	1.27 ± 0.06 a	1.58 ± 0.24 ab	1.72 ± 0.31 b	2.30 ± 0.31 c		
Ethyl lactate (mg/L) ^A^	4.88 ± 0.18 a	7.16 ± 0.67 b	9.40 ± 0.42 c	11.63 ± 0.76 d	154.636 [35]	Fruity, buttery [35]
Ethyl octanoate ^A^	**307.31 ± 24.64 a**	**475.80 ± 20.62 b**	**556.63 ± 137.48 b**	**807.86 ± 121.61 c**	0.58 [36]	Sweet, floral, fruity, banana, pear, brandy [35]
Ethyl 7-octenoate ^B^	6.90 ± 0.47 a	18.55 ± 2.25 b	19.85 ± 1.40 b	22.8 ± 0.38 c		
Ethyl 3-hydroxybutyrate ^B^	2.98 ± 0.23 a	3.28 ± 0.28 a	3.77 ± 0.60 ab	4.78 ± 1.57 b		
Ethyl nonanoate ^A^	1.26 ± 0.19 b	0.88 ± 0.24 a	1.43 ± 0.25 b	1.14 ± 0.20 ab	0.85 [37]	Fatty, oily, cognac, nut-like odor; oily, fatty-fruity taste [37]
Ethyl 2-hydroxy-4-methylpentanoate (mg/L) ^B^	1.49 ± 0.32 a	2.98 ± 0.06 b	3.58 ± 0.10 c	4.33 ± 0.12 d		
Ethyl furoate ^B^	0.46 ± 0.01 a	0.77 ± 0.01 b	0.91 ± 0.03 c	1.00 ± 0.10 d		
Ethyl decanoate ^A^	**270.21 ± 24.26 a**	**259.4 ± 54.90 a**	**367.62 ± 97.66 b**	**495.84 ± 15.03 c**	0.2 [38]	Brandy, fruity, grape [35]
Ethyl benzoate ^B^	3.45 ± 0.08 a	5.52 ± 0.31 b	6.39 ± 0.41 c	7.42 ± 0.54 d		
Diethyl succinate (mg/L) ^A^	0.38 ± 0.03 a	0.68 ± 0.03 b	0.96 ± 0.03 c	1.15 ± 0.07 d	1200 [35]	Fruity, melon [35]
Ethyl 9-decenoate ^B^	100.11 ± 6.33 a	129.61 ± 13.08 ab	155.78 ± 34.29 b	212.55 ± 24.71 c		
Ethyl phenylacetate ^B^	4.18 ± 0.14 a	6.01 ± 0.39 b	6.97 ± 0.34 c	7.53 ± 0.41 d		
Ethyl dodecanoate ^A^	82.31 ± 6.78 a	83.8 ± 10.88 a	116.89 ± 5.56 b	145.15 ± 24.82 c	1.5 [35]	Oily, fatty, fruity [35]
Ethyl myristate ^B^	70.20 ± 2.75 a	93.73 ± 9.75 b	118.03 ± 5.58 c	133.89 ± 15.4 d		
Diethyl malate ^B^	5.56 ± 1.36 a	12.76 ± 4.01 b	11.71 ± 1.94 b	13.61 ± 2.48 b		
Ethyl 3-hydroxy tridecanoate ^B^	8.13 ± 0.22 a	17.57 ± 1.86 b	23.04 ± 2.78 c	26.12 ± 1.92 c		
Ethyl pentadecanoate ^B^	0.39 ± 0.11 a	0.72 ± 0.10 a	1.17 ± 0.19 b	1.33 ± 0.47 b		
Ethyl palmitate ^B^	26.02 ± 1.41 a	32.35 ± 2.79 a	39.07 ± 3.24 b	44.8 ± 6.86 b		
Ethyl 9-hexadecenoate ^B^	70.38 ± 4.17 c	20.42 ± 1.84 a	22.68 ± 1.60 ab	26.32 ± 1.74 b		
Ethyl hydrogen succinate ^B^	16.34 ± 4.59 a	43.27 ± 12.6 b	35.55 ± 3.68 b	43.71 ± 4.93 b		
Total ethyl esters (mg/L)	6.89 ± 0.31 a	10.92 ± 0.67 b	13.81 ± 0.38 c	17.07 ± 0.88 d		
Other esters						
Isobutyl caproate ^B^	0.11 ± 0.01 a	0.15 ± 0.03 a	0.25 ± 0.05 b	0.29 ± 0.08 b		
Isoamyl hexanoate ^A^	0.89 ± 0.08 a	1.30 ± 0.10 ab	1.70 ± 0.46 b	2.22 ± 0.36 c	-	Fruity, banana, apple, pineapple, green [39]
Isobutyl octanoate ^B^	1.26 ± 0.15 a	1.41 ± 0.19 a	3.07 ± 0.54 b	3.70 ± 0.84 b		
Isoamyl octanoate ^B^	2.42 ± 0.08 a	2.46 ± 0.26 a	3.22 ± 0.60 b	3.83 ± 0.34 c		
Isopentyl decanoate ^B^	20.75 ± 1.17 a	19.87 ± 1.79 a	24.12 ± 3.47 a	30.03 ± 3.46 b		
Total other esters	25.42 ± 1.34 a	25.19 ± 2.22 a	32.35 ± 4.95 b	40.06 ± 4.80 c		
Total esters (mg/L)	20.17 ± 1.38 a	26.93 ± 3.66 b	29.30 ± 2.24 b	38.49 ± 1.75 c		
**Higher Alcohols**						
Isobutanol (mg/L) ^A^	74.69 ± 2.07 a	**91.84 ± 6.81 b**	**104.38 ± 4.45 c**	**118.51 ± 8.49 d**	75 [36]	Alcohol, nail polish [35]
1-Butanol (mg/L) ^A^	0.42 ± 0.01 a	0.58 ± 0.05 ab	0.71 ± 0.04 b	1.15 ± 0.29 c	150 [35]	Medicinal, phenolic [35]
Isoamyl alcohol (mg/L) ^A^	**146.81 ± 4.49 a**	**197.55 ± 7.01 b**	**204.03 ± 7.77 bc**	**213.25 ± 9.72 c**	60 [36]	Solvent, sweet, alcohol, nail polish [35]
4-Methyl-1-pentanol ^A^	12.44 ± 0.80 a	18.63 ± 0.96 b	18.64 ± 1.25 b	18.81 ± 1.17 b	50 [35]	Almond, toasted [35]
3-Methyl-1-pentanol ^B^	40.04 ± 1.03 a	49.13 ± 1.01 b	46.85 ± 4.20 b	42.34 ± 2.52 a		
1-Hexanol ^A^	8.70 ± 0.30 a	11.77 ± 0.64 b	12.87 ± 1.05 b	12.52 ± 1.04 b	1.1 [35]	Herbaceous, grass, woody [35]
*cis*-3-Hexenol ^B^	17.44 ± 3.77 a	21.38 ± 1.87 ab	25.61 ± 2.35 bc	29.17 ± 3.22 c		
1-Heptanol ^A^	4.93 ± 0.55 a	11.24 ± 0.71 b	15.80 ± 1.67 c	22.05 ± 2.49 d	0.2 [35]	Oily [35]
2-Nonanol ^A^	2.77 ± 0.06 a	4.22 ± 0.18 b	4.07 ± 0.14 b	4.18 ± 0.25 b	0.058 [40]	Waxy green creamy citrus orange cheese fruity [39]
*levo*-2,3-Butanediol (g/L) ^A^	**1.12 ± 0.16 a**	**2.46 ± 0.45 b**	**1.60 ± 0.38 a**	**2.70 ± 0.30 b**	150 [41]	Fruity, sweet, butter [35]
1-Octanol ^A^	0.38 ± 0.03 a	0.66 ± 0.05 b	0.90 ± 0.02 c	1.12 ± 0.05 d	0.8 [35]	Jasmine, lemon [35]
1-Nonanol ^B^	0.59 ± 0.05 a	0.89 ± 0.09 b	1.09 ± 0.07 c	1.27 ± 0.07 d		
2-Undecanol ^B^	2.67 ± 0.22 a	4.08 ± 0.30 b	3.87 ± 0.53 b	4.42 ± 0.57 b		
1-Decanol ^A^	0.18 ± 0.01 a	0.90 ± 0.09 b	1.24 ± 0.19 c	1.96 ± 0.11 d	0.4 [35]	Fatty, waxy, floral, orange, sweet, clean ,watery [39]
Benzyl alcohol ^A^	21.34 ± 3.06 a	30.14 ± 2.28 b	33.04 ± 3.14 b	41.99 ± 2.65 c	200 [35]	Roasted, sweet, fruity [35]
2-Phenylethanol (mg/L) ^A^	**36.79 ± 2.52 a**	**49.63 ± 2.84 b**	**52.74 ± 2.63 bc**	**56.37 ± 4.55 c**	14 [38]	Roses, honey [35]
*meso*-2,3-Butanediol (mg/L) ^A^	**416.52 ± 72.13 a**	**708.72 ± 184.22 b**	**592.29 ± 98.02 ab**	**988.36 ± 108.14 c**	150 [41]	Fruity, sweet, butter [35]
1-Dodecanol ^A^	tr	tr	0.03 ± 0.05 a	0.17 ± 0.12 b	1 [40]	Flowery [40]
Total alcohols (g/L)	1.35 ± 0.15 a	2.75 ± 0.44 c	1.91 ± 0.37 b	3.03 ± 0.29 c		
**Acids**						
Acetic acid ^A^	538.66 ± 26.36 a	639.49 ± 58.90 ab	747.59 ± 67.60 bc	873.73 ± 144.99 c	200 [40]	Acid, fatty [40]
Isobutyric acid (mg/L) ^A^	**13.39 ± 0.21 a**	**14.44 ± 0.95 a**	**16.78 ± 0.72 b**	**17.76 ± 0.96 b**	2.3 [38]	Rancid, butter, cheese [42]
3-Methylbutanoic acid ^B^	14.47 ± 2.05 a	14.68 ± 4.29 a	13.31 ± 3.60 a	17.74 ± 4.31 a		
2-Methylbutanoic acid (mg/L) ^B^	2.57 ± 0.19 a	3.00 ± 0.13 b	3.03 ± 0.16 b	3.01 ± 0.24 b		
Butyric acid ^A^	306.88 ± 13.70 a	392.08 ± 33.81 b	451.87 ± 28.89 bc	520.62 ± 71.77 c	2.3 [35]	Rancid, butter, cheese [35]
Hexanoic acid (mg/L) ^A^	**1.04 ± 0.02 a**	**1.14 ± 0.02 b**	**1.21 ± 0.02 c**	**1.26 ± 0.04 d**	0.42 [38]	Cheese, fatty [35]
Octanoic acid (mg/L) ^A^	**1.46 ± 0.09 a**	**1.87 ± 0.09 b**	**2.20 ± 0.18 c**	**2.52 ± 0.28 d**	0.5 [38]	Rancid, cheese, fatty acid [35]
7-Octenoic acid ^B^	38.50 ± 7.64 a	50.84 ± 4.46 b	63.27 ± 4.73 c	74.96 ± 10.95 c		
*n*-Decanoic acid ^A^	715.48 ± 25.30 a	698.68 ± 32.51 a	757.30 ± 53.56 ab	816.75 ± 65.02 b	1 [35]	Fatty, rancid [35]
9-Decenoic acid ^B^	258.77 ± 11.74 a	285.13 ± 11.52 ab	321.52 ± 21.32 bc	356.93 ± 36.48 c		
*Total acids (mg/L)*	20.33 ± 0.26 a	22.52 ± 0.96 b	25.57 ± 1.01 c	27.20 ± 1.38 d		
**Aldehydes**						
Nonanal ^A^	0.01 ± 0.00 a	0.01 ± 0.00 a	0.01 ± 0.00 c	0.02 ± 0.00 b	0.015 [35]	Green, slightly pungent [35]
Furfural ^B^	0.04 ± 0.01 a	0.08 ± 0.01 b	0.10 ± 0.01 c	0.12 ± 0.02 d		
Decanal ^A^	2.12 ± 0.43 a	3.01 ± 0.64 ab	3.81 ± 0.53 bc	4.20 ± 0.82 c	0.01 [35]	Grassy, orange skin-like [40]
5-Methylfurfural ^B^	9.57 ± 0.76 a	12.41 ± 1.43 b	17.09 ± 1.69 c	23.25 ± 2.05 d		
Dodecanal ^B^	tr	tr	0.14 ± 0.11 a	0.38 ± 0.19 b		
*Total aldehydes*	11.73 ± 1.00 a	15.51 ± 1.36 b	21.15 ± 2.20 c	27.97 ± 2.68 d		
**Ketones**						
2-Nonanone ^B^	tr	0.66 ± 0.62	tr	tr		
2-Undecanone ^B^	3.71 ± 0.53 a	4.12 ± 0.66 ab	3.81 ± 0.59 ab	4.84 ± 0.86 b		
*Total ketones*	3.71 ± 0.53 a	4.78 ± 1.22 a	3.81 ± 0.59 a	4.84 ± 0.86 a		
**Terpene derivatives**						
Linalool ^A^	4.92 ± 0.15 a	7.08 ± 0.43 b	6.72 ± 0.38 b	6.62 ± 0.42 b	0.025 [35]	Flowery, muscat [35]
4-Terpineol ^B^	6.92 ± 0.17 a	8.15 ± 0.34 b	8.56 ± 0.28 b	9.39 ± 0.50 c		
β-*cis*-Farnesene ^B^	9.13 ± 0.55 a	13.84 ± 0.93 b	15.85 ± 0.71 c	17.35 ± 1.39 c		
*p*-Menth-1-en-8-ol ^A^	15.00 ± 0.67 a	19.27 ± 1.14 b	21.54 ± 1.09 c	23.20 ± 1.16 c	0.25 [43]	Pine, terpene, lilac, citrus, woody, floral [39]
(*Z,E*)-α-Farnesene ^B^	2.41 ± 0.07 a	2.88 ± 0.37 b	3.07 ± 0.37 bc	3.53 ± 0.25 c		
Naphthalene ^A^	0.01 ± 0.00 a	0.01 ± 0.00 a	0.01 ± 0.00 b	0.01 ± 0.00 b	-	-
1,1,6-Trimethyl-1,2-dihydronaphtalene ^B^	0.14 ± 0.01 a	0.14 ± 0.02 a	0.19 ± 0.02 b	0.24 ± 0.04 c		
β-Bisabolene ^B^	3.13 ± 0.17 a	3.74 ± 0.41 ab	4.07 ± 0.51 b	4.94 ± 0.52 c		
α-Farnesene ^B^	3.80 ± 0.18 a	5.03 ± 0.63 b	5.61 ± 0.85 b	6.85 ± 0.50 c		
Citronellol ^A^	8.12 ± 0.41 a	11.85 ± 0.80 b	12.39 ± 0.79 b	12.76 ± 0.74 b	0.1 [35]	Rose [35]
α-Caryophyllene ^B^	2.99 ± 0.22 a	3.35 ± 0.47 ab	3.91 ± 0.51 b	4.84 ± 0.42 c		
β-Damascenone ^B^	1.63 ± 0.04 a	2.11 ± 0.14 b	2.29 ± 0.14 c	2.39 ± 0.08 c		
Geranylacetone ^A^	3.66 ± 0.28 a	5.32 ± 0.59 b	7.17 ± 0.96 c	8.84 ± 0.75 d	0.06 [44]	Fresh, green, fruity, waxy, rose, woody, magnolia, tropical [39]
*trans*-Nerolidol ^A^	26.89 ± 1.94 a	38.39 ± 5.00 b	38.78 ± 3.47 b	37.05 ± 3.55 b	0.25 [45]	Rose, apple, green, citrus, waxy, woody [45]
2,3-Dihydrofarnesol ^B^	7.51 ± 0.41 a	14.04 ± 2.04 b	12.47 ± 2.43 b	13.82 ± 1.19 b		
Farnesol^B^	0.17 ± 0.06 a	0.44 ± 0.09 c	0.38 ± 0.07 bc	0.29 ± 0.02 b		
*Total terpene derivatives*	41.66 ± 1.06 a	55.12 ± 2.36 b	60.00 ± 2.71 c	65.28 ± 3.40 d		
**Others**						
Acetoin (mg/L) ^A^	39.52 ± 2.31 ab	35.53 ± 2.32 a	42.32 ± 3.66 b	42.55 ± 4.79 b	150 [35]	Buttery, fatty [35]
*p*-Cymene ^A^	0.31 ± 0.04 a	0.26 ± 0.06 a	0.44 ± 0.07 b	0.44 ± 0.04 b	0.0114 [45]	Citrus, green [45]
2-Acetylfuran ^B^	0.58 ± 0.05 a	1.28 ± 0.12 b	1.59 ± 0.08 c	1.88 ± 0.17 d		
γ-Butyrolactone ^B^	42.49 ± 2.78 a	64.45 ± 7.64 ab	81.77 ± 18.78 b	115.05 ± 20.11 c		
Methionol ^B^	0.04 ± 0.00 a	0.06 ± 0.00 c	0.05 ± 0.00 bc	0.05 ± 0.01 ab		
4-Hydroxy-2-methylacetophenone ^B^	6.51 ± 0.37 a	9.27 ± 0.71 b	10.32 ± 0.79 bc	11.05 ± 1.04 c		
Total others (mg/L)	39.57 ± 2.31 ab	35.61 ± 2.32 a	42.41 ± 3.61 b	42.68 ± 4.80 b		

^a^ Volatiles with superscript ^A^ are identified by comparing their mass spectrum with the available standard; Volatiles with superscript ^B^ were tentatively identified by comparing their mass spectrum with mass spectral database and retention index from literatures; ^b^ Volatile concentration is expressed as μg/L; “tr” represents “trace amount. ”Concentration over odor threshold (esters over OAV values of 0.1) is displayed in bold. Data are the mean ± standard deviation of triplicate tests. Different letters in each row indicate significant differences at *p* ≤ 0.05.

## Supplementary Materials

Supplementary materials can be accessed at: http://www.mdpi.com/1420-3049/22/1/52/s1.
