# Supplementary material for: Dibasic Ammonium Phosphate Application Enhances Aromatic Compound Concentration in Bog Bilberry Syrup Wine
Source: Molecules. 2016 Dec 29;22(1):52. doi: 10.3390/molecules22010052 (PMC6155706; doi:10.3390/molecules22010052)
Supplement: Supplementary file 1 [file molecules-22-00052-s001.pdf]

Supplementary Materials: Dibasic Ammonium Phosphate Application Enhances Aromatic Compound Concentration in Bog Bilberry Syrup Wine

Shao-Yang Wang, Yi-Qing Li, Teng Li, Hang-Yu Yang, Jie Ren, Bo-Lin Zhang and Bao-Qing Zhu

Table S1. Physicochemical indexes of bog bilberry syrup wine supplemented with 0 mg/L and 30 mg/L dibasic ammonium phosphate (DAP).

Physicochemical Index	Wine with Dibasic Ammonium Phosphate (mg/L) [a]	
	0	30
Alcohol (%, vol)	2.7 ± 0.3	6.9 ± 0.2
Total Sugar (g/L)	134.23 ± 7.41	52.70 ± 3.40
Reducing Sugar (g/L)	125.10 ± 6.52	46.05 ± 2.45
pH	3.13 ± 0.01	3.13 ± 0.01
Total Acidity (g/L)	9.42 ± 0.03	9.31 ± 0.05

[a] Data are the mean ± standard deviation of triplicate tests.

Table S2. Odor activity value (OAV) and aroma series of main volatile compounds in bog bilberry syrup wine supplemented with different amounts of dibasic ammonium phosphate (DAP).

Volatile Compounds	Odor Activity Value (OAV) of Volatile in Wine with DAP (mg/L) [b]				Aroma Series [a]
	60	90	120	150	
**Esters**					
Acetate esters					
Ethyl acetate	1.04 ± 0.09 a	1.27 ± 0.25 a	1.21 ± 0.17 a	1.68 ± 0.10 b	1, 5, 6 [36]
Isoamyl acetate	1.61 ± 0.10 a	1.57 ± 0.33 a	1.75 ± 0.21 ab	2.22 ± 0.46 b	3, 5 [36]
Phenethyl acetate	0.05 ± 0.00 a	0.09 ± 0.00 b	0.13 ± 0.01 c	0.16 ± 0.02 d	2 [36]
Ethyl esters					
Ethyl butanoate	1.97 ± 0.20 a	2.45 ± 0.58 ab	2.05 ± 0.34 ab	2.62 ± 0.25 b	5 [36]
Ethyl hexanoate	1.97 ± 0.08 a	2.69 ± 0.40 b	2.46 ± 0.38 ab	2.57 ± 0.56 ab	5 [36]
Ethyl octanoate	0.53 ± 0.04 a	0.82 ± 0.04 b	0.96 ± 0.24 b	1.39 ± 0.21 c	2, 3, 4 [36]
Ethyl decanoate	1.35 ± 0.12 a	1.30 ± 0.27 a	1.84 ± 0.49 b	2.48 ± 0.08 c	5 [36]
**Higher Alcohols**					
Isobutanol	1.00 ± 0.03 a	1.22 ± 0.09 b	1.39 ± 0.06 c	1.58 ± 0.11 d	5 [36]
Isoamyl alcohol	2.45 ± 0.07 a	3.29 ± 0.12 b	3.40 ± 0.13 bc	3.55 ± 0.16 c	1, 7, 4 [35]
levo-2,3-Butanediol	7.50 ± 1.04 a	16.39 ± 2.99 b	10.66 ± 2.52 a	18.01 ± 1.99 b	6, 7, 4 [35]
2-Phenylethanol	2.63 ± 0.18 a	3.55 ± 0.20 b	3.77 ± 0.19 bc	4.03 ± 0.32 c	2 [35]
meso-2,3-Butanediol	2.78 ± 0.48 a	4.72 ± 1.23 b	3.95 ± 0.65 ab	6.59 ± 0.72 c	5, 7, 4 [35]
**Acids**					
Isobutyric acid	5.82 ± 0.09 a	6.28 ± 0.41 a	7.29 ± 0.31 b	7.72 ± 0.42 b	4 [36]
Hexanoic acid	2.49 ± 0.04 a	2.70 ± 0.04 b	2.88 ± 0.04 c	2.99 ± 0.09 d	4 [36]
Octanoic acid	2.92 ± 0.19 a	3.73 ± 0.17 b	4.39 ± 0.36 c	5.04 ± 0.55 d	4 [36]

[a] Aroma series: 1 = solvent, 2 = floral, 3 = sweet, 4 = fatty, 5 = fruity, 6 = balsamic. 7 = caramel; [b] Data are the mean ± standard deviation of triplicate tests. Different letters in each row indicate significant differences at $p \leq 0.05$.

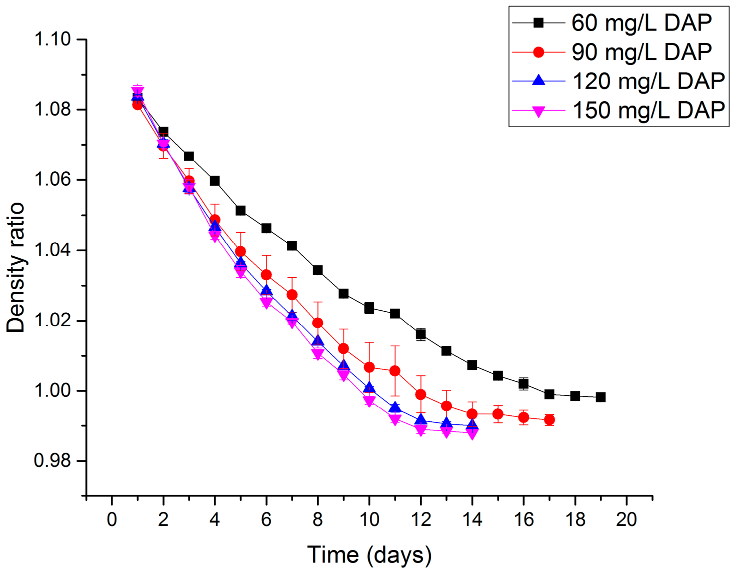

Figure S1. Density ratio of bog bilberry syrup wine fermentation under different DAP amounts addition.
